# Simple and Efficient Targeting of Multiple Genes Through CRISPR-Cas9 in *Physcomitrella patens*

**DOI:** 10.1534/g3.116.033266

**Published:** 2016-09-08

**Authors:** Mauricio Lopez-Obando, Beate Hoffmann, Carine Géry, Anouchka Guyon-Debast, Evelyne Téoulé, Catherine Rameau, Sandrine Bonhomme, Fabien Nogué

**Affiliations:** * Institut Jean-Pierre Bourgin, National Institute for Agricultural Research, AgroParisTech, Centre National de la Recherche Scientifique, Université Paris-Saclay, 78000 Versailles, France; † Université Pierre et Marie Curie, Sorbonne Universités, 75005 Paris, France

**Keywords:** CRISPR-Cas9, moss, multiple gene targeting, butenolide receptor, KAI2, AP2/ERF transcription factor

## Abstract

Powerful genome editing technologies are needed for efficient gene function analysis. The CRISPR-Cas9 system has been adapted as an efficient gene-knock-out technology in a variety of species. However, in a number of situations, knocking out or modifying a single gene is not sufficient; this is particularly true for genes belonging to a common family, or for genes showing redundant functions. Like many plants, the model organism *Physcomitrella patens* has experienced multiple events of polyploidization during evolution that has resulted in a number of families of duplicated genes. Here, we report a robust CRISPR-Cas9 system, based on the codelivery of a CAS9 expressing cassette, multiple sgRNA vectors, and a cassette for transient transformation selection, for gene knock-out in multiple gene families. We demonstrate that CRISPR-Cas9-mediated targeting of five different genes allows the selection of a quintuple mutant, and all possible subcombinations of mutants, in one experiment, with no mutations detected in potential off-target sequences. Furthermore, we confirmed the observation that the presence of repeats in the vicinity of the cutting region favors deletion due to the alternative end joining pathway, for which induced frameshift mutations can be potentially predicted. Because the number of multiple gene families in *Physcomitrella* is substantial, this tool opens new perspectives to study the role of expanded gene families in the colonization of land by plants.

The bryophyte *Physcomitrella patens* has been used as a model plant to bridge the knowledge gap in early land plant gene function (Strotbek *et al.* 2013). Genetic studies have been stimulated by the publication of its genome sequence (Rensing *et al.* 2008), and the availability of various functional genetic tools, *e.g.*, single gene knockouts in *P. patens* are obtained efficiently by gene targeting thanks to the high rate of homologous recombination (HR), and the ease of transformation of protoplasts (Schaefer 2001; Schaefer and Zryd 1997). Furthermore, the haploid status of most of the *P. patens* life cycle, and the stem cell potential of its cells (Prigge and Bezanilla 2010), facilitate gene and mutant studies in *P. patens*. Genome analysis shows that recent gene and genome duplication events have contributed to the expansion of several gene families in *P. patens* (Zimmer *et al.* 2013). Contrary to single mutants, the isolation of multiple mutants needs laborious and time-consuming crosses or retransformation procedures with novel knockout constructs. RNA interference (RNAi) has been developed as an alternative technology for multiple gene targets (Bezanilla *et al.* 2005; Nakaoka *et al.* 2012), and, using tandem RNAi, it was possible to silence eight genes simultaneously (Vidali *et al.* 2009). However, establishing RNAi stable lines can be challenging. Moreover, in most cases, RNAi seems to favor gene knockdown rather than knockout in this moss (Burkart *et al.* 2015; Nakaoka *et al.* 2012).

Recently, the use of sequence-specific nucleases, and, particularly, the clustered regularly interspaced short palindromic repeats (CRISPR) and CRISPR associated proteins (Cas) systems (Makarova *et al.* 2015), have been adapted for gene targeting in different organisms (Wright *et al.* 2016). The type II CRISPR-Cas9 system was the first one engineered to mediate genome editing of eukaryotic cells (Jinek *et al.* 2012). This system used an engineered single guide RNA (sgRNA) in which the 20 bp comprising the CRISPR RNA (crRNA) upstream of a protospacer adjacent motif (PAM; NGG or NAG for Cas9) was fused to the *trans*-activating crRNA (tracrRNA) sequence of the Cas9 protein. In plants, CRISPR-Cas9 mediated genome editing of single and multiple gene targets has been shown for several vascular plants (Ceasar *et al.* 2016; Ma *et al.* 2016; Zhang *et al.* 2016). In nonvascular plants, the CRISPR-Cas9 system has been used in *Marchantia polymorpha* to target the *AUXIN RESPONSE FACTOR 1* (ARF1) gene following *Agrobacterium*-mediated transformation (Sugano *et al.* 2014), and in *P. patens* to target the *ADENINE PHOSPHORIBOSYL TRANSFERASE* (*PpAPT*) gene by protoplast transformation (Collonnier *et al.* 2016). However, multiple gene editing has not yet been shown for nonvascular plants. In order to test whether CRISPR-Cas9 technology could be used for efficient targeting of multiple genes in *P. patens*, we focused on targeting the *P. patens KARRIKIN INSENSITIVE 2 LIKE* (*PpKAI2L*) gene family. These genes are homologs of *KARRIKIN INSENSITIVE 2/HYPOSENSITIVE TO LIGHT* (*KAI2/HTL*) and *DWARF 14* (*D14*) vascular plant genes, encoding receptors and candidate receptors of butenolide compounds such as strigolactones or karrikins (Lopez-Obando *et al.* 2016). The 13 genes of this family are split into two clades, (i) and (i.i–iii), that were targeted separately. Furthermore, we spread out this approach to target a small family of four members in the APETALA 2/ERE binding factor (AP2/ERF) transcription factor gene family (Mizoi *et al.* 2012). The results presented here indicate that the CRISPR-Cas9 system is a simple and powerful tool for the generation of multiple mutations in the moss *P. patens* that probably surpasses existing tools for multiple gene targeting in this organism.

## Materials and Methods

### Cloning and sgRNA plasmid preparation

Coding sequences of *PpKAI2L* and AP2/ERF genes were used to search for CRISPR RNA (crRNA) preceded by a PAM motif of the *Streptococcus* *pyogenes* Cas9 (NGG or NAG) using the webtool CRISPOR V1 against *P. patens* genome Phytozome V9 (http://crispor.tefor.net/crispor.py). crRNAs close to the translation start codon (ATG) with high specificity score, and few predicted off-targets, were selected for cloning (Supplemental Material, Table S1 and Table S2). A fragment of 500 bp containing the snRNA U3 or U6 promoter (Collonnier *et al.* 2016) followed by a sgRNA, and flanked by AttB recombinant sites, was synthetized chemically as gBlocks (Integrated DNA Technologies) (Figure S1). sgRNAs are composed of 20 bp of the crRNA fused to 83 bp of the *S*. *pyogenes* tracrRNA scaffold (Mali *et al.* 2013). Each fragment was cloned into a pUC57 (GenScript) or pDONR207 (Invitrogen) backbone. Plasmids were amplified in *Escherichia coli* DH5α, and purified using Nucleobond PC100 or Nucleospin plasmid kit (Macherey-Nagel).

### Moss culture and transformation

Wild-type *P. patens* Gransden strain was used for transformation experiments. Moss was grown in chambers set at 60% humidity with 16 hr of light (quantum irradiance of 80 µmol m^−2^ s^−1^) at 24°, and 8 hr of dark at 22°. Plants were grown on PpNH_4_ medium (PpNO_3_ medium supplemented with 2.7 mM NH_4_-tartrate). For protoplast isolation, two rounds of 7-d-old moss protonema tissue were used as starting material. Protoplast isolation and transformation followed a previously described protocol (Ashton *et al.* 1979; Schaefer and Zryd 1997) with minor modifications (Figure S2). Protoplasts were transformed with a total of 20 µg of circular DNA divided as follow: 8–10 µg of the pAct-Cas9 plasmid that contains a Cas9 expression cassette with the rice Actin 1 promoter, shown to be active in *P. patens* (Horstmann *et al.* 2004), and a codon-optimized version of Cas9 from *S. pyogenes* fused to a SV40 nuclear localization (Mali *et al.* 2013), and a mix of 10–12 µg of sgRNA plasmids. The amount of each sgRNA plasmid in the mix was obtained by dividing the total amount of sgRNA plasmids (10–12 µg) by the number of sgRNA plasmids used for each transformation. For experiments using antibiotic selection, the pBNRF plasmid, which contains a 35S::neoR cassette for resistance to G418 (Schaefer *et al.* 2010), was added. The amount of pBNRF plasmid was the same as that of a sgRNA in the respective experiments. Plants were regenerated on cellophane disks, and plated on PpNH_4_ medium containing 0.33 M of mannitol for 1 wk. Then, plants on cellophane disks were transferred to a new PpNH_4_ plate for 1 wk without selection, or with 50 mg/l of G418 (Duchefa) selection when pBNRF plasmid was added. The pBNRF plasmid was used here for transient selection. The rationale for this assay was the capacity, in *P. patens*, of transformed DNA to replicate episomally allowing antibiotic selection to be maintained during multiple cycles of divisions (Ashton *et al.* 2000; Murén *et al.* 2009; Schaefer *et al.* 1991). Plants were put onto a new PpNH_4_ plate for 1 wk, and then individualized; 1 or 2 wk later, a sample of each clone was taken for high-throughput DNA extraction.

### DNA extraction and PCR screening of on-target mutations

Gametophore and protonema tissue from 3- to 4-wk-old moss clones were dispensed into 96-well Microtube Rack plates (National Scientific Supply) containing two steel balls per well. Frozen or lyophilized moss tissue was ground using a TissueLyserII system (Qiagen). The high-throughput DNA extraction fast method was performed as follows: 150 μl of extraction buffer (0.2 M Tris-HCl pH 7.5, 0.25 M NaCl, 0.025 M EDTA, 0.5% SDS) was added to each well. After mixing and 5 min of centrifugation (3220 × *g*), 100 μl of supernatant was transferred to a 96 v-shape well microplate (Greiner bio-one). DNA was precipitated by adding 100 μl of isopropanol and centrifuging for 15 min at 3220 × *g*. The supernatant was discarded, and precipitated DNA was cleaned by adding 200 μl of ethanol 75%, and centrifuged 15 min at 3220 × *g*. The ethanol was discarded, and precipitated DNA was dried for 20 min at 37°. Finally, DNA was dissolved in 80–100 μl of TE buffer. PCR reactions (50 µl) were performed using 1 μl of extracted DNA solution as template. Primers for PCR gel shift analysis were designed to obtain 150–200 bp fragments surrounding the on-target crRNA sequence (Table S3). Primer3plus software (http://www.bioinformatics.nl/cgi-bin/primer3plus/primer3plus.cgi) was used to design primers (Untergasser *et al.* 2007). PCR fragment shifts were evaluated in 3% agarose TBE gel-electrophoresis. When a shift was detected, the fragment was sequenced. Sequences were aligned by CodonCode Aligner V5.1.5 from LI-COR. Alignments were curated manually to find mutations around the PAM sequence of the corresponding on-target genomic sequence. Clones with a confirmed mutation were also sequenced for all on-target genomic sequences.

### Off-target screening

Regions surrounding potential off-target sites predicted by the CRISPOR software (Table S1) were sequenced in the mutants. Primers flanking the corresponding off-target sequence were designed using Primer3plus software (Table S2). Sequences were aligned by CodonCode Aligner V5.1.5 from LI-COR. Alignments were curated manually to detect potential mutations around the PAM sequence of the corresponding off-target sites.

### Data availability

The authors state that all data necessary for confirming the conclusions presented in the article are represented fully within the article.

## Results and Discussion

### Efficient CRISPR-Cas9 multiple gene targeting

The web-based CRISPOR program was used to search and select the crRNA, and to predict potential off-target sites (Hsu *et al.* 2013). Using this tool, a unique crRNA that would target two or more *PpKAI2L* genes at the same time was not found. Hence, a highly specific crRNA was selected for each *PpKAI2L* gene separately (Table S1). The tracrRNA sequence was fused to each specific crRNA, to generate sgRNA under the control of *P. patens* U3 or U6 promoters (Figure S1). The PEG-mediated transformation protocol was adapted to deliver a mix of the sgRNA plasmids along with the plasmid containing the *S. pyogenes* human codon-optimized Cas9 (*Sp*-hCas9) into *P. patens* protoplasts (Figure S2). We initially selected five out of the 13 *PpKAI2L* genes (clade i) as targets, these genes being related most closely to the Arabidopsis *KAI2/HTL* gene (Lopez-Obando *et al.* 2016). Two independent transformation experiments, I and II, used multiple gene-specific constructs targeting *PpKAI2L-A to D* and *PpKAI2L-A* to *-E*, respectively. These experiments were done without selection, and successful on-target events were detected on gel, looking for shift of PCR fragments; 472 and 477 independent moss clones were screened in experiments I and II, respectively (Figure S3). When a PCR fragment shift was detected for any targeted gene in one clone, the PCR fragments of all targeted genes were sequenced. A total of 57 (12% of the total) and 26 (5.4% of the total) clones were identified as mutants in experiments I and II, respectively. Because, in our conditions, the PCR fragment shift screen cannot detect insertion or deletion below 5 bp, we sequenced PCR products of 105 clones chosen randomly in experiment I. Six new mutants were detected. Therefore, the PCR fragment shift approach failed to detect about 10% (6/63) of the total number of mutants obtained in this experiment.

**Figure 1:**
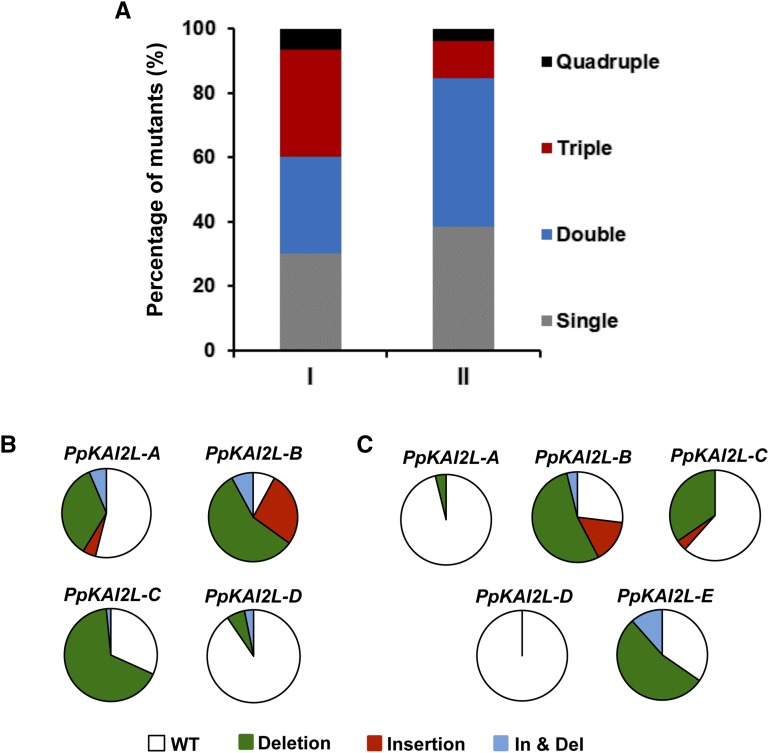
Multiplex gene targeting in *P. patens PpKAI2L* clade i gene family using a CRISPR-Cas9 system without selection pressure. (A) Percentage of single and multiple mutants obtained in experiments I (*n* = 63) and II (*n* = 26), targeting the *PpKAI2L* genes of clade i. Experiment I uses a mix of four sgRNA plasmids targeting *PpKAI2L-A* to –*D*. Experiment II uses a mix of five sgRNA plasmids targeting *PpKAI2L-A* to -*E*. (B) Distribution of deletion, insertion, and insertion-deletion (In & Del) ontarget events found for each sgRNA in experiment I. (C) Distribution of deletion, insertion, and insertion-deletion (In & Del) on-target events found for each sgRNA in experiment II.

**Figure 2:**
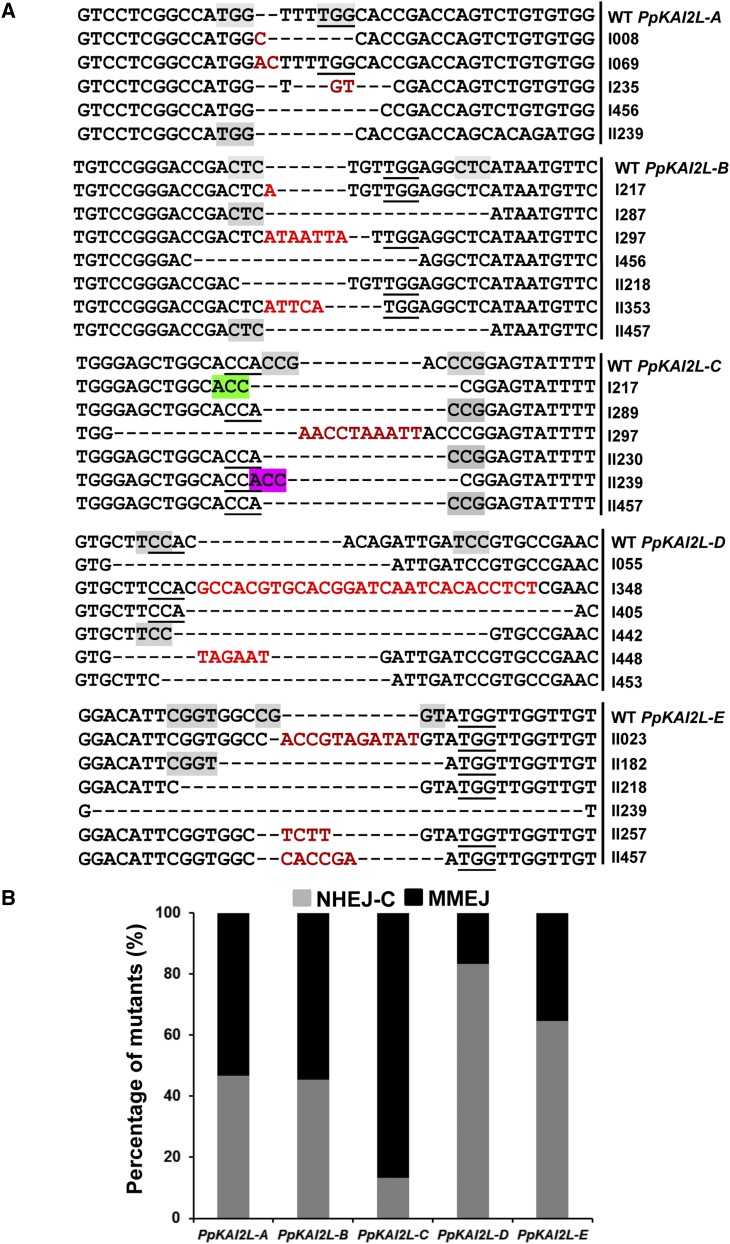
Mutations detected by sequence analysis and putative associated DNA repair mechanisms. (A) Examples of insertions and deletions in the *PpKAI2L-B* gene of experiments I and II. Clones obtained from the experiments I and II were called I#clone and II#clone, respectively. The protospacer adjacent motif (PAM) upstream of the crRNA is underlined in the sequence. Inserted sequences are shown in red. Microhomology sequences likely used for DNA repair are indicated with gray shading. (B) Percentage of mutations likely using the classical nonhomologous end joining (C-NHEJ), and the microhomology-mediated end joining (MMEJ), DNA repair mechanisms in the mutant population of experiments I and II. Data show the proportion of C-NHEJ *vs.* MMEJ for each gene of the *PpKAI2L* clade i targeted by their corresponding sgRNA used in this study.

Single and combinations of multiple mutants were obtained in these experiments (Figure 1A, Table S4, and Table S5). The sgRNAs showed different mutagenic activities and percentages of frameshift mutations, ranging from 20 to 95% of total mutations found for each sgRNA (Table S6). All sgRNAs induced insertion, deletion and insertion-deletion mutation types, deletions being more abundant, as observed previously in other organisms including *P. patens* where CRISPR-Cas9 was tested (Figure 1, B and C, Figure 2A) (Collonnier *et al.* 2016; Sugano *et al.* 2014). The efficiency of each sgRNA to generate mutations in the on-target region was different comparing one to each other. In these experiments, the sgRNAs targeting *PpKAI2L-A* and *PpKAI2L-D* were found to be less efficient (Figure 1, B and C). As the guanine content and GC percentage were similar for all five crRNAs, and because the sgRNA targeting PpKAI2L-A, *i.e.*, the less efficient sgRNA, is also the one with the highest GC content, these features cannot explain the difference in mutation efficiency observed. However, many other characteristics could explain these differences, among which efficiency of codelivery of plasmids, accessibility of the CRISPR complexes to the on-target regions (Horlbeck *et al.* 2016), or internal sgRNA interactions (Thyme *et al.* 2016).

Two types of DNA repair mechanisms could explain the observed mutations: the classical nonhomologous end joining (C-NHEJ) pathway, and the microhomology-mediated end joining (MMEJ) pathway, the latter relying on 2–16 bp microhomology regions to repair double-strand breaks in DNA (Sfeir and Symington 2015). Such microhomology regions were noted when sequencing the moss mutations, and examples are given in Figure 2A, where mutants from experiments I and II have been pooled. The proportion of use of C-NHEJ and MMEJ differs from one sgRNA to another (Figure 2B). As an example, mutations induced by the sgRNA targeting *PpKAI2L-B* showed an overrepresentation of a deletion of 12 bp, probably resulting from a MMEJ-mediated repair using two CTC repeats (38/77), while for the sgRNA targeting *PpKAI2L-C*, a similar MMEJ repair mechanism could involve CCG (24/53) and ACC (19/53) repeats (Figure 2A). In contrast, in the case of the sgRNAs targeting *PpKAI2L-D* and *PpKAI2*L-E, most of the mutations observed do not involve microhomologies, and thus C-NHEJ seems to have been the preferential repair pathway (Figure 2B). These results are in agreement with what has been observed previously in other eukaryotes, and with predicted patterns of microhomology repair (Bae *et al.* 2014). In order to evaluate off-target activity for each sgRNA, top potential off-target loci predicted by CRISPOR (Table S1) were amplified with surrounding primers and sequenced in 10 mutants. No mutations were detected in these potential off-target sequences for any of the tested mutants. The most probable reason for off-target inactivity of Cas9 is the presence of mismatches in the seed region of all crRNAs (1–12 bases after PAM). Indeed, this point was found to be critical for sgRNA specificity in previous studies on human cells (Hsu *et al.* 2013; Pattanayak *et al.* 2014). Because the crRNAs designed here were highly specific to the targeted locus, this confirms that such specificity allows minimizing off-target activity of the Cas9 protein in *P. patens*. These results are also in accordance with previous studies showing a lack of off-target activity of Cas9 in vascular plants (Feng *et al.* 2014; Pan *et al.* 2016) and in *P. patens* (Collonnier *et al.* 2016). However, it will be important to conduct a comprehensive genome analysis of mutant individuals, as noncanonical off-targets caused by DNA bulges (insertions) have been reported in human cells (Lin *et al.* 2014).

### Transient selection allows 100% of multiplex gene editing

**Figure 3:**
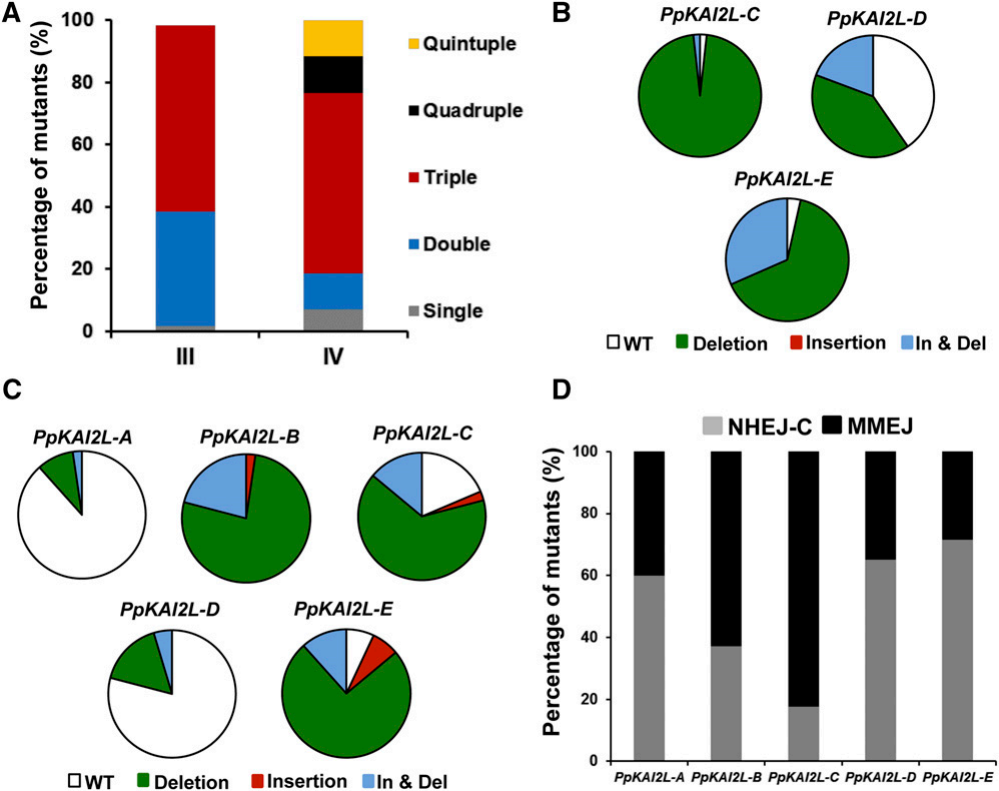
Multiplex gene targeting in *P. patens PpKAI2L* clade i gene family using a CRISPR-Cas9 system with transient selection. (A) Percentage of single and multiple mutants obtained in experiments III (*n* = 57) and IV (*n* = 43) targeting the *PpKAI2L* genes of clade i. Experiment III used a mix of three sgRNA plasmids targeting *PpKAI2L-C* to -*E* genes along with pBNRF plasmid. Experiment IV used a mix of five sgRNA targeting *PpKAI2L-A* to -*E* genes along with pBNRF plasmid. (B) Distribution of deletion, insertion, and insertion-deletion (In & Del) on-target events found for each sgRNA in experiment III. (C) Distribution of deletion, insertion, and insertion-deletion (In & Del) on-target events found for each sgRNA in experiment IV. (D) Percentage of mutations likely using the classical nonhomologous end joining (C-NHEJ) and the microhomology-mediated end joining (MMEJ) DNA repair mechanisms in the mutant population of experiments III and IV. Data show the proportion of C-NHEJ *vs.* MMEJ for each gene of the *PpKAI2L* clade i targeted by their corresponding sgRNA used in this study.

In an attempt to increase the efficiency of our multiple gene knock-out tool, we set up a strategy aiming at selecting transiently transformed protoplasts. For this purpose, we took advantage of the possibility in *Physcomitrella* of episomal replication of the transformed DNA (Murén *et al.* 2009), and used the pBNRF plasmid containing a 35S::neoR cassette for transient resistance of the transformed protoplasts to geneticin. This approach relies on the fact that (1) circular plasmids do not integrate stably into *Physcomitrella* chromosomes; and (2) due to the high copy number of plasmids, transformed protoplasts take up all constructs present. We performed two new cotransformation experiments, III and IV, with sgRNAs targeting *PpKAI2L-C* to -*E* and *PpKAI2L-A* to –*E*, respectively, along with the *Sp*-hCas9 and the pBNRF plasmids. After transient selection on geneticin, we picked 57 and 43 clones for analysis in experiments III and IV, respectively. PCR fragments around the targeted region for each gene were obtained and sequenced directly. Strikingly, in both experiments, we identified 98 and 100% of mutated clones respectively. This indicates that transient selection removes most, if not all, of the clones that were not cotransformed, and thus not subjected to both pBNRF expression and CRISPR-Cas9 activity. Compared to experiments I and II, we found fewer single mutants and more multiple mutants, including quintuple mutants that were not found in the absence of selection (Figure 3A). However, the ratio of mutation types, the number of frameshift mutations, and the proportion of the two NHEJ pathways (C-NHEJ *vs.* MMEJ) observed for each sgRNA were similar to those observed in experiments I and II (Figure 3, B–D, Table S4, and Table S5). In these experiments, as in experiments I and II, both sgRNAs targeting *PpKAI2L-A* and *PpKAI2L-D* genes were again found less active to induce mutations (Figure 3, B and C).

### High efficiency of the CRISPR-Cas9 mediated multiplex gene editing is confirmed for two other gene families

**Figure 4:**
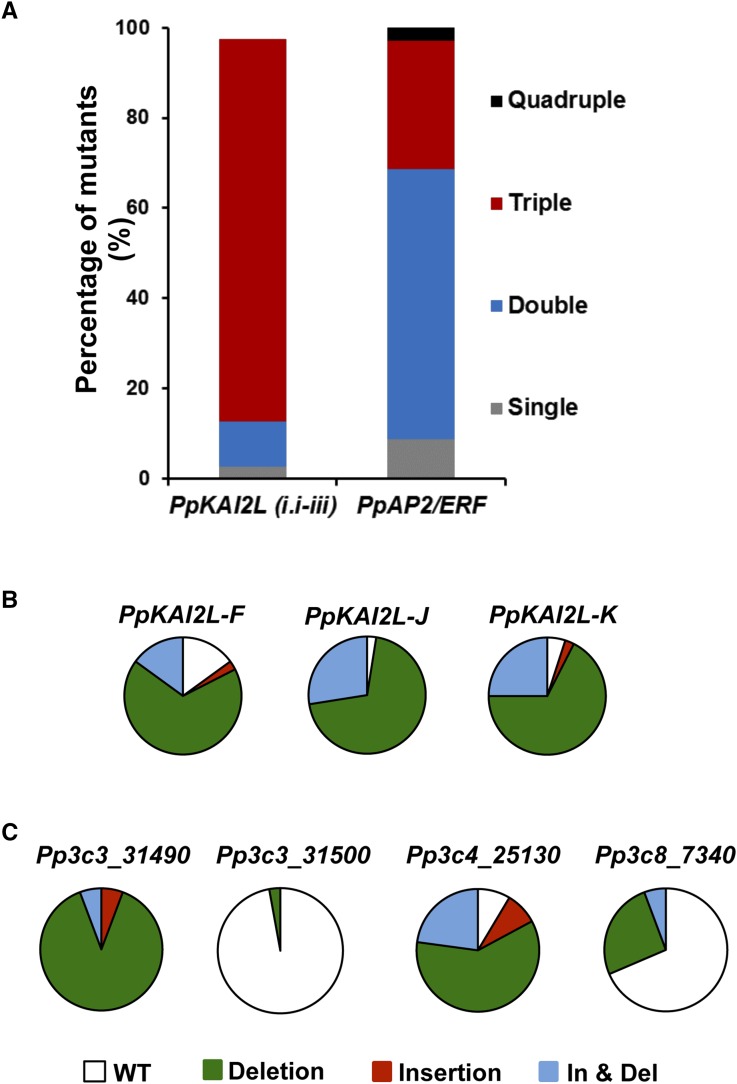
Features of mutations in *PpKAI2L* clade i.i–iii and *PpAP2/ERF* genes using a CRISPR-Cas9 system. (A) Percentage of single and multiple mutants obtained using a mix of three sgRNA plasmids targeting the *PpKAI2L-F*, *PpKAI2L-J*, and *PpKAI2L-K* genes of clade i.i–iii along with pBNRF plasmid (*n* = 40), and that obtained from a mix of four sgRNA targeting *AP2/ERF* transcription factor genes along with pBNRF plasmid (*n* = 35). (B) Distribution of deletion, insertion, and insertion-deletion (In- & Del) on-target events found for each sgRNA targeting the *PpKAI2L-F*, *PpKAI2L-J*, and *PpKAI2L-K* genes of clade i.i–iii. (C) Distribution of deletion, insertion, and insertion-deletion (In & Del) on-target events found for each sgRNA targeting the *PpAP2/ERF Pp3c3_31490*, *Pp3c3_31500*, *Pp3c4_25130*, and *Pp3c8_7340*.

In order to test the robustness of this multiple gene knock-out strategy based on transient expression of the CRISPR-CAS9 module, we evaluated the efficiency of multiplex gene editing of the *PpKAI2LF*, *PpKAI2LJ*, and *PpKAI2LK* genes from the *PpKAI2L* clade i.i–iii (Lopez-Obando *et al.* 2016), and of four genes (*Pp3c3_31490*, *Pp3c3_31500*, *Pp3c4_25130*, and *Pp3c8_7340*) belonging to the AP2/ERF transcription factor family (Mizoi *et al.* 2012) (Table S7). After transient selection, 40 and 35 clones were evaluated for mutations in the three and four genes, respectively. In these populations, we found 97.5 and 100% of mutants, respectively. Furthermore, similar results as in previous experiments regarding the proportion of mutants, and the type of mutations, were found (Figure 4, A–C). Interestingly, as was found in experiments III and IV, where transient selection was applied, a high rate of multiple mutants *vs.* single mutants was observed, and mutants for all targeted copies were obtained (Figure 4A). In the experiment targeting *PpKAI2L-F*, *PpKAI2L-J*, and *PpKAI2L-K*, we also evaluated the off-target activity of Cas9 in seven triple and one double mutants, and did not find any mutation for several CRISPOR-predicted off-target sites (Table S8).

In conclusion, we have shown that, combined with the selection of transiently transformed clones, CRISPR-Cas9 technology can be used as an efficient tool to accelerate research in expanded gene families of the bryophyte *P. patens*. Furthermore, the approach of multiple gene editing shown here is simpler and more straightforward than the existing gene targeting tools for *P. patens*. The presence of repeats in the vicinity of the cutting region favors deletion due to the MMEJ pathway, for which induced frameshift mutations can be potentially predicted. Therefore, local microhomology, and resulting deletions creating a frameshift, should be considered during the design of the crRNA when a knockout mutant is desired. The lack of detection of off-target activity for sgRNAs tested here suggests that the CRISPR-Cas9 system may have low off-target activity in *P. patens*. Because the number of multiple gene families in *Physcomitrella* is substantial, this tool opens important perspectives for the functional analysis of these genes in this model plant, including those likely involved in colonization of land by plants. Finally, this strategy opens new prospects for the knock-out of genes belonging to multigene families in plants amenable to protoplast transformation and regeneration.

## 

## Supplementary Material

Supplemental Material
