# Supplementary material for: Simple and Efficient Targeting of Multiple Genes Through CRISPR-Cas9 in Physcomitrella patens
Source: G3 (Bethesda). 2016 Sep 8;6(11):3647–53. doi: 10.1534/g3.116.033266 (PMC5100863; doi:10.1534/g3.116.033266)
Supplement: Supplemental Material [file supp_6_11_3647__index.html]

Simple and Efficient Targeting of Multiple Genes Through CRISPR-Cas9 in Physcomitrella patens — Supplemental Material 

# Simple and Efficient Targeting of Multiple Genes Through CRISPR-Cas9 in *Physcomitrella patens*

## Supplemental Material for Lopez-Obando, *et al*, 2016

**Files in this Data Supplement:**

- Figure S1 - Schematic representation of *P. patens* protoplast co-transformation with CRISPR-Cas9 multiplex system. (.pdf, 11 KB)
- Table S7 - Target genes and crRNA sequences for members of the *PpKAI2L* gene family clade i.i-iii and *PpAP2/ERF* transcription factors gene family. (.pdf, 84 KB)
- Table S8 - Selected predicted off-targets against members of the *PpKAI2L* gene family clade i.i-iii for the analysis of off-target activity. (.pdf, 85 KB)
- Figure S2 - Schematic representation of constructs leading to the expression of synthetic sgRNA. (.pdf, 80 KB)
- Figure S3 - Example of fragment shift of *PpKAI2L-B* PCR products from regenerated clones after agarose gel electrophoresis. (.pdf, 433 KB)
- Table S1 - Features of selected CRISPR RNA (crRNA) against members of the *PpKAI2L* gene family clade i. (.pdf, 78 KB)
- Table S2 - Target genes and crRNA sequences for members of the *PpKAI2L* gene family clade i.i-iii and *PpAP2/ERF* transcription factors gene family. (.pdf, 84 KB)
- Table S3 - Primers used in this study. (.pdf, 70 KB)
- Table S4 - List of mutants obtained using sgRNAs against *PpKAI2L-A, PpKAI2L-B, PpKAI2L-C* and *PpKAI2L-D* clade i genes without selection. (.pdf, 117 KB)
- Table S5 - List of mutants obtained using sgRNAs against *PpKAI2L-A, PpKAI2L-B, PpKAI2L-C, PpKAI2L-D* and *PpKAI2L-E* clade i genes without selection. (.pdf, 93 KB)
- Table S6 - Percentage of frameshift mutations per gene in experiments I to IV targeting *PpKA2L* genes of clade i. (.pdf, 185 KB)
