# Supplementary material for: Simple and Efficient Targeting of Multiple Genes Through CRISPR-Cas9 in Physcomitrella patens
Source: G3 (Bethesda). 2016 Sep 8;6(11):3647–53. doi: 10.1534/g3.116.033266 (PMC5100863; doi:10.1534/g3.116.033266)
Supplement: Supplemental Material [file supp_g3.116.033266_FigureS1.pdf]

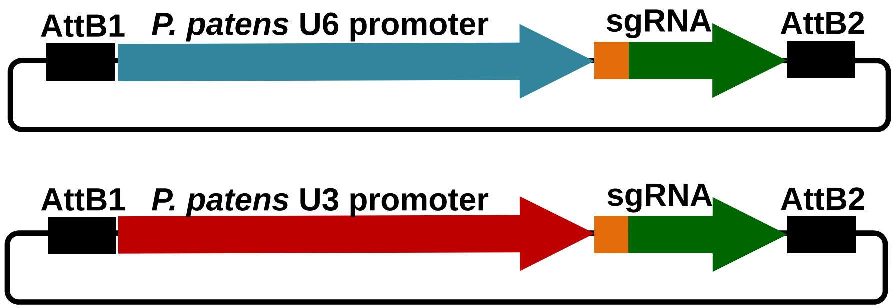

Figure S1. Schematic representation of *P. patens* protoplast co-transformation with CRISPR-Cas9 multiplex system.

The sgRNA is composed of a CRISPR RNA (crRNA) sequence in orange (20 nucleotides, specific for each gene) and the trans-activating crRNA (tracrRNA) sequence in green (common to all sgRNAs). AttB regions are shown in black, *P. patens* U3 promoter is shown in red and *P. patens* U6 promoter is shown in blue.
