# Supplementary material for: Simple and Efficient Targeting of Multiple Genes Through CRISPR-Cas9 in Physcomitrella patens
Source: G3 (Bethesda). 2016 Sep 8;6(11):3647–53. doi: 10.1534/g3.116.033266 (PMC5100863; doi:10.1534/g3.116.033266)
Supplement: Supplemental Material [file supp_g3.116.033266_TableS8.pdf]

Table S8. Selected predicted off-targets against members of the *PpKAI2L* gene family clade i.i-iii for the analysis of off-target activity.

On-target gene	Predicted off-targets sequence[a,b]	Off-target annotation[c]
PpKAI2L-F	AGAGGCGTTTCCACACCGTC*CGG*	OffT1*: 5' UTR of Pp3c15_18460 locus
	AAACGTATTTCCACATCCTG*TGG*	OffT2*: Exon of Pp3c16_12790
PpKAI2L-J	ACGTTCCGAGCCCTCAGGCA*AGG*	OffT2*: 3' UTR of Pp3c15_11920 locus
	ACGTTGAGAGCCGTGATGTA*GGG*	OffT5*: Exon of Pp3c15_4920
PpKAI2L-K	AGGTGGCCAAGTACATGGTG*AGG*	OffT1*: Exon of Pp3c5_23900
	AAATGGAGCATTACATAGTA*TGG*	OffT2*: 3' UTR of Pp3c2_2130 locus
	AAATGGCGAAATCCATTGTA*AGG*	OffT3*: Exon of Pp3c15_5520

[a]: Protospacer adjacent motif (PAM) sequence preceded by crRNA sequence is shown in italics and underlined. SS; Specificity score data from CRISPOR (Hsu *et al.*, 2013),
[b]: Mismatch of off-target sequence with respect to the crRNA sequence is shown in red,
[c]: Identifier and annotation of genomic elements from Phytozome V11 - *P. patens* genome V3.3
*Off-targets for which the activity of the sgRNA was measured by sequencing PRC fragments of the surrounding predicted site in eight mutants
