# Supplementary material for: Simple and Efficient Targeting of Multiple Genes Through CRISPR-Cas9 in Physcomitrella patens
Source: G3 (Bethesda). 2016 Sep 8;6(11):3647–53. doi: 10.1534/g3.116.033266 (PMC5100863; doi:10.1534/g3.116.033266)
Supplement: Supplemental Material [file supp_g3.116.033266_FigureS3.pdf]

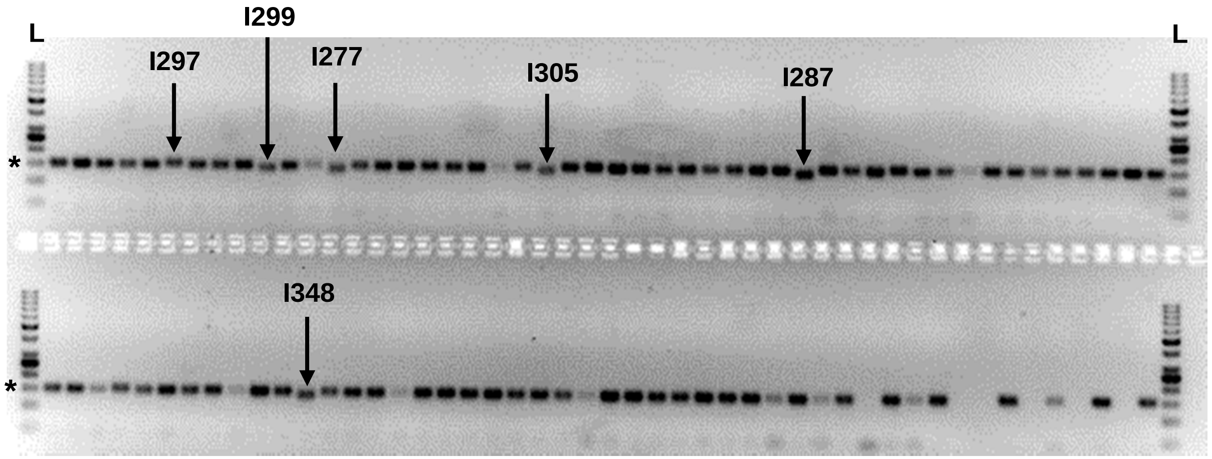

Figure S3. Example of fragment shift of *PpKAI2L-B* PCR products from regenerated clones after agarose gel electrophoresis.

Fragments correspond to 150 bp surrounding the on-target region of *PpKAI2L-B* gene of clones obtained from the co-transformation with the mix of four sgRNA targeting *PpKAI2L-A*, *PpKAI2L-B*, *PpKAI2L-C* and *PpKAI2L-D* genes of the experiment I. Electrophoresis was carried out using 3% agarose gel. Clones showing a fragment shift compared to the majority of clones (presumably non-mutated plants) are indicated with arrows. Asterisk indicates the 150 bp fragment of the 50 bp gene ruler DNA ladder (L) (Thermo Scientific).
