# Supplementary material for: Simple and Efficient Targeting of Multiple Genes Through CRISPR-Cas9 in Physcomitrella patens
Source: G3 (Bethesda). 2016 Sep 8;6(11):3647–53. doi: 10.1534/g3.116.033266 (PMC5100863; doi:10.1534/g3.116.033266)
Supplement: Supplemental Material [file supp_g3.116.033266_TableS1.pdf]

Table S1. Features of selected CRISPR RNA (crRNA) against members of the *PpKAI2L* gene family clade i.

On-target gene[a]	crRNA sequence and SS (%)[b]	Predicted off-targets sequence[b,c]	Off-target annotation[a]
Pp3c2_19340 (*PpKAI2L-A*)	GTTGTCCTCGGCCATGGTTT*TGG* (99)	CTTGACCTGGTCCATGGTTT*TGG*	OffT1*: Intergenic between Pp3c22_8523 - Pp3c22_8540
		GTAGTCCTAAGCCATGGGTT*TGG*	OffT2*: Exon of Pp3c10_1460 (PpKAI2L-F)
		GTAGTGCTCAGCCATGGATT*TGG*	OffT3*: Exon of Pp3c1_18010 (PpKAI2L-K)
		GTAGTCCTCATCCATGGGTT*CGG*	OffT4*: Exon of Pp3c19_19860
		GTGGTACTAGGCCATGGGTT*TGG*	OffT5: Exon of Pp3c12_8770 (PpKAI2L-I)
		GTGGTCCACGGCCATGGCGT*TGG*	OffT6: 5'UTR of Pp3c20_10270 locus
		TTTTTCCTCGGCATTGGTTT*TGG*	OffT7: Intergenic between Pp3c22_14060 - Pp3c22_14160
		GTTGTTCTTGGCGATGGGTT*GGG*	OffT8: 3'UTR of Pp3c1_24440
		GTTGGCCTCGGCCTTGCTGT*CGG*	OffT9: Exon of Pp3c19_4690
		CTTGTCCTCGGGCGTGGCTT*CGG*	OffT10: nf
		GTTGTCCTCGACGTTGGTTC*TGG*	OffT11: Exon of Pp3c12_14550
		GTTGTCCTTGGCTTGGGTTT*GGG*	OffT12: Intergenic between Pp3c20_19870 - Pp3c20_19940
Pp3c14_6110 (*PpKAI2L-B*)	GTGTCCGGGACCGACTCTGT*TGG* (100)	GTGTACGGGTCCGACGGTGT*CGG*	OffT1: Exon of Pp3c5_25610
		GTGTACGGGTCCGACGGTGT*CGG*	OffT2: Exon of Pp3c5_25430
		CTGTCCGGTACCCGCTCTGT*AGG*	OffT3*: Exon of Pp3c11_17570
		GTGTCCAGGACGGACACTTT*GGG*	OffT4: Exon of Pp3c1_4090
Pp3c25_5350 (*PpKAI2L-C*)	GCTAAAATACTCCGGGTCGG*TGG* (99)	GCTGAAGTACTCGGGGTCTG*TGG*	OffT1*: Exon of Pp3c6_10610 (PpKAI2L-D)
		GCTAAAGTACTCCGGATCTG*TGG*	OffT2*: Exon of Pp3c2_19340 (PpKAI2L-A)
		GCCAAAATGCTCCGTGTCGC*AGG*	OffT3*: Exon of Pp3c20_4150
Pp3c6_10610 (*PpKAI2L-D*)	TCGGCACGGATCAATCTGTG*TGG* (98)	TTGGCACAGATCAATCCGTG*TGG*	OffT1*: Exon of Pp3c25_5350 (PpKAI2L-C)
		TCGGCACGGATCAGTCGGTG*TGG*	OffT2*: Exon of Pp3c5_16420 (PpKAI2L-E)
		TCGACATGGGTCAAGCTGTG*AGG*	OffT3*: Exon of Pp3c2_19340 (PpKAI2L-A)
		TCCGCACGGGGCAATGTGTG*AGG*	OffT4: 3'UTR of Pp3c7_22220
		TTGGCACCGACCAGTCTGTG*TGG*	OffT5: Exon of Pp3c6_29320
Pp3c5_16420 (*PpKAI2L-E*)	TGGACATTCGGTGGCCGGTA*TGG* (99)	TGAAAATTCTGTGGCCGGTA*AGA*	OffT1*: 5'UTR of Pp3c26_10400
		TGGACATTCGGTGGGGGGTA*TGG*	OffT2*: Exon of Pp3c16_8770
		TGGGCATTCTGTGTCTGGTA*TGG*	OffT3*: Exon of Pp3c6_10610 (PpKAI2L-D)
		TGGACACTCGGTGTCAGGCA*TGG*	OffT4*: Exon of Pp3c25_5350 (PpKAI2L-C)

[a]: Identifier and annotation of genomic elements from Phytozome V11 - *P. patens* genome V3.3, nf: not found,
[b]: Protospacer adjacent motif (PAM) sequence preceded by crRNA sequence is shown in italics and underlined. SS; Specificity score data from CRISPOR (Hsu *et al.*, 2013),
[c]: Mismatch of off-target sequence with respect to the crRNA sequence is shown in red,
*Off-targets for which the activity of the sgRNA was measured by sequencing PRC fragments of the surrounding predicted site in 10 mutants.
