# Supplementary material for: Simple and Efficient Targeting of Multiple Genes Through CRISPR-Cas9 in Physcomitrella patens
Source: G3 (Bethesda). 2016 Sep 8;6(11):3647–53. doi: 10.1534/g3.116.033266 (PMC5100863; doi:10.1534/g3.116.033266)
Supplement: Supplemental Material [file supp_g3.116.033266_TableS2.pdf]

Table S2. Target genes and crRNA sequences for members of the *PpKAI2L* gene family clade i.i-iii and *PpAP2/ERF* transcription factors gene family.

On-target gene[a]	crRNA sequence and SS (%)[b]
Pp3c10_1460 (*PpKAI2L-F*)	AGACGTATTTCCACACCGTC*TGG* (99)
Pp3c4_32050 (*PpKAI2L-J*)	ACGTTGTGAGCCTTCAGGTA*AGG* (100)
Pp3c1_18010 (*PpKAI2L-K*)	AAGTGGCGAATTACATGGTA*CGG* (99)
Pp3c3_31490	GCCAAAGAGTGGGAAACCGGT*GGG* (99)
Pp3c3_31500	AGTCGTCTCCTCATTCTTCA*CGG* (99)
Pp3c4_25130	GCAGCTGTAACTCTACCGTC*AGG* (99)
Pp3c8_7340	GCTGCGATGGAGGTTAGGGC*AGG* (99)

[a]: Identifier and name (in brackets) of genes from Phytozome V11 - *P. patens* genome V3.3

[b]: Protospacer adjacent motif (PAM) sequence preceded by crRNA sequence is shown in italics and underlined. SS; Specificity score data from CRISPOR is shown in brackets (Hsu *et al.*, 2013)
