# Supplementary material for: Simple and Efficient Targeting of Multiple Genes Through CRISPR-Cas9 in Physcomitrella patens
Source: G3 (Bethesda). 2016 Sep 8;6(11):3647–53. doi: 10.1534/g3.116.033266 (PMC5100863; doi:10.1534/g3.116.033266)
Supplement: Supplemental Material [file supp_g3.116.033266_TableS3.pdf]

Table S3. Primers used in this study.

Name	5' to 3' sequence
LA_sgRNA_LCoffT2-LD_offT3_F	CATCGGGATATGTCTTACTCTCC
LA_sgRNAR	GAGCTTATAATCATCAACGAGGTG
LB_sgRNAF	GTAACATTGCGCACCTTGTG
LB_sgRNAR	GCAAGAACCACCAGCTCTTC
LC_sgRNAF	GGAAACACGTCATTCCTCATC
LC_sgRNAR	ACCTCCAGTTCGTCCAAAAT
LD_sgRNA_LCoffT1_ LEoffT3F	ATGGAGGAAGGACCAACACTCT
LD_sgRNA_LCoffT1_ LEoffT3R	AGAAGCGGGGTTGGAATAGT
LE_sgRNAF	CTCGAGATACTCTACCCTTCACG
LE_sgRNA_LD_offT2_R	GTACCTTGGAGAAGCCGAGA
LAoffT1_F	CTGCAGCACCACCTAATGAA
LAoffT1_R	CTTTGATGCTGTATGTTACCAACC
LAoffT2_F	TGCAGTCCCACAATGTGATAAT
LAoffT2_R_LK_s	TCATGATGCAAAACATCGCAAGAG
LAoffT3_F_LK_sgRNAF	ATCTCGCGGTTTTCTGTGAT
LAoffT3_R_LK_sgRNAR	GCAGTGCGAACTGAGTGATG
LAoffT4_F	GCTACCGCTTGCCAGAATAG
LAoffT4_R	TTCTGGAGTGCTTTGACGAA
LBoffT3_F	GGTTCGGGAGAAGAACTGGT
LBoffT3_R	CATGTGGACGTGGTGATAGC
LBoffT4_F	GTGAGGTTTGGGATCGAGTG
LBoffT4_R	CTTCCAGTTCTTGCGACCAT
LCoffT2-LD_offT3_R	GTAACACGCTGCGCAAATC
LCoffT3_F	AGTGGCGCTAGTGGTGCTAT
LCoffT3_R	CCGAGCTGGGCTAATAATCA
LD_offT1-LEoffT4_F	ATGGATGAGCTACCATCACTC
LD_offT1-LEoffT4_R	TCAACAAGACTCCAGATTCCCAG
LD_offT2_F	GTGAGTGAGTTGGCGATGTC
LEoffT1_F	TACTTTGCCCTGCGTACGTT
LEoffT1_R	TACGCCTCAACCACACGTTA
LEoffT2_F	GGGTCCGGCATACTTAGCTT
LEoffT2_R	ATCGTTTCAAAAACGGCAAG
LF_sgRNAF	GGAGATAGAACCATGCAGTCCCACAATGTGATAAT
LF_sgRNAR	TCCACCTCCGGATCCTGATGCAAAACATCGCAAGAG
LF_qR293_seq	CAACCGATCATTCCAGACAC
LJ_sgRNAF	GGAGATAGAACCATGATTCAGAAATTGACACCACCTGAGAC
LJ_sgRNAR	TCCACCTCCGGATCCGGCAGCCCAGAGCAAGGC
LJ_qR538_seq	CCATCGCCTTAGGTACAAAACC
LF_OffT1F	GGCCTGCTAGGAAAGTAACAA
LF_OffT1R	CATGTGACACAAGAATGTGTGG
LF_OffT2F	GGCAGGTCTTTCCCTTGAAT
LF_OffT2R	ATGGTCGGAGACGATCAAAA
LJ_OffT2F	TAACCATTCGCCAAGTAGCC
LJ_OffT2R	CGCAAATTCATATGTATCGTAAGC
LJ_OffT5F	TCCACGTATTGCCGCTTTAT
LJ_OffT5R	AATGATTCAAACCATGCAACC
LK_OffT1F	ATCCTACGGCAGTCTGAACG
LK_OffT1R	ATTGTCGCACAATGCTCCAT
LK_OffT2F	AAGTTCCCATCCTCCTACATGA
LK_OffT2R	AACCATATTCCATCATCTTTGACA
LK_OffT3F	AATTGCGACCTCGGTATCAG
LK_OffT3R	GAACCATGCATCAATCTTGC
Pp003G122800_1	GGATCCAATCAGCCCACTTGTGT
Pp003G122800_lw	ACGAAGGCGATGAGTGAAGT
Pp003G122900_1	GGATCCTACAAAGGCCAGCCATC
Pp003G122900_lw	ATACGCTCTTGCAGCCATCT
Pp004G095800_7	CCGAACCTTCACTTGGTGTT
Pp004G095800_lw	CGATTGATTGATGTCGAACG
Pp008G027400_1	AAGCTTGGCAAGTGTGATGAGATT
Pp008G027400_lw	AAGCTTGGCAAGTGTGATGAGATT
