# Supplementary material for: Simple and Efficient Targeting of Multiple Genes Through CRISPR-Cas9 in Physcomitrella patens
Source: G3 (Bethesda). 2016 Sep 8;6(11):3647–53. doi: 10.1534/g3.116.033266 (PMC5100863; doi:10.1534/g3.116.033266)
Supplement: Supplemental Material [file supp_g3.116.033266_TableS4.pdf]

Table S4. List of mutants obtained using sgRNAs against *PpKAI2L-A, PpKAI2L-B, PpKAI2L-C* and *PpKAI2L-D* clade i genes without selection.

Mutant Name	Target gene[a]	Type of mutation (bp)[b]	Microhomology motif[c]
I220	*PpKAI2L-A*	Deletion (6)	TGG
I006	*PpKAI2L-B*	Deletion (12)	CTC
I022	*PpKAI2L-B*	Deletion (12)	CTC
I025	*PpKAI2L-B*	Deletion (12)	CTC
I054	*PpKAI2L-B*	Insertion (1)	nf
I122	*PpKAI2L-B*	Deletion (12)	CTC
I127	*PpKAI2L-B*	Deletion (12)	CTC
I131	*PpKAI2L-B*	Deletion (18)	TGT
I186	*PpKAI2L-B*	Insertion (16) and deletion (3)	nf
I234	*PpKAI2L-B*	Deletion (12)	CTC
I246	*PpKAI2L-B*	Insertion (4)	nf
I247	*PpKAI2L-B*	Deletion (12)	CTC
I278	*PpKAI2L-B*	Insertion (1)	nf
I287	*PpKAI2L-B*	Deletion (12)	CTC
I485	*PpKAI2L-B*	Deletion (12)	CTC
I411	*PpKAI2L-B*	Deletion (10)	nf
I441	*PpKAI2L-B*	Insertion (4)	nf
I404	*PpKAI2L-C*	Deletion (5)	nf
I449	*PpKAI2L-C*	Deletion (5)	CCG
I245	*PpKAI2L-A*<br>*PpKAI2L-B*	Deletion (17)<br>Deletion (12)	nf<br>CTC
I277	*PpKAI2L-A*<br>*PpKAI2L-B*	Deletion (6)<br>Deletion (12)	TGG<br>CTC
I344	*PpKAI2L-A*<br>*PpKAI2L-B*	Insertion (2) and deletion (19)<br>Insertion (4)	nf<br>nf
I444	*PpKAI2L-A*<br>*PpKAI2L-C*	Insertion (1) and deletion (18)<br>Deletion (4)	nf<br>ACC
I456	*PpKAI2L-A*<br>*PpKAI2L-C*	Deletion (8)<br>Deletion (7)	nf<br>ACC
I118	*PpKAI2L-B*<br>*PpKAI2L-C*	Deletion (12)<br>Deletion (7)	CTC<br>ACC
I195	*PpKAI2L-B*<br>*PpKAI2L-C*	Deletion (2)<br>Deletion (5)	nf<br>CCG
I266	*PpKAI2L-B*<br>*PpKAI2L-C*	Deletion (12)<br>Deletion (5)	CTC<br>CCG
I297	*PpKAI2L-B*<br>*PpKAI2L-C*	Insertion (7) and deletion (2)<br>Deletion (5)	nf<br>CCG
I364	*PpKAI2L-B*<br>*PpKAI2L-C*	Insertion (4)<br>Deletion (5)	nf<br>CCG
I385	*PpKAI2L-B*<br>*PpKAI2L-C*	Deletion (12)<br>Deletion (20)	CTC<br>GAG
I389	*PpKAI2L-B*<br>*PpKAI2L-C*	Insertion (12)<br>Deletion (5)	nf<br>CCG
I398	*PpKAI2L-B*<br>*PpKAI2L-C*	Deletion (12)<br>Deletion (5)	CTC<br>CCG
I409	*PpKAI2L-B*<br>*PpKAI2L-C*	Deletion (12)<br>Deletion (5)	CTC<br>CCG
I416	*PpKAI2L-B*<br>*PpKAI2L-C*	Insertion (1)<br>Deletion (4)	nf<br>ACC
I422	*PpKAI2L-B*<br>*PpKAI2L-C*	Insertion (10)<br>Deletion (5)	nf<br>CCG
I454	*PpKAI2L-B*<br>*PpKAI2L-C*	Deletion (18)<br>Deletion (5)	nf<br>CCG

[a]: Genes without mutation (WT) are not shown
[b]:Number of base pairs in brackets
[c]: nf: not found

Table S4 Continued

Mutant Name	Target gene[a]	Type of mutation (bp)[b]	Microhomology motif[c]
I465	*PpKAI2L-B*	Deletion (12)	nf
	PpKAI2L-C	Deletion (4)	ACC
I469	*PpKAI2L-B*	Deletion (12)	CTC
	PpKAI2L-C	Deletion (7)	ACC
I008	*PpKAI2L-A*	Insertion (1)	nf
	PpKAI2L-B	Insertion (1)	nf
	PpKAI2L-C	Deletion (5)	CCG
I041	*PpKAI2L-A*	Deletion (6)	TGG
	PpKAI2L-B	Deletion (18)	TGT
	PpKAI2L-C	Deletion (5)	CCG
I069	*PpKAI2L-A*	Insertion (2)	nf
	PpKAI2L-B	Insertion (10)	nf
	PpKAI2L-C	Deletion (5)	CCG
I126	*PpKAI2L-A*	Deletion (6)	TGG
	PpKAI2L-B	Insertion (1)	nf
	PpKAI2L-C	Deletion (5)	CCG
I202	*PpKAI2L-A*	Insertion (19)	nf
	PpKAI2L-B	Deletion (12)	CTC
	PpKAI2L-C	Deletion (5)	CCG
I206	*PpKAI2L-A*	Deletion (6)	TGG
	PpKAI2L-B	Deletion (12)	CTC
	PpKAI2L-C	Deletion (4)	ACC
I217	*PpKAI2L-A*	Insertion (2) and Deletion (5)	nf
	PpKAI2L-B	Insertion (1)	nf
	PpKAI2L-C	Deletion (5)	CCG
I235	*PpKAI2L-A*	Insertion (2) and deletion (8)	nf
	PpKAI2L-B	Insertion (8) and deletion (10)	nf
	PpKAI2L-C	Deletion (5)	CCG
I289	*PpKAI2L-A*	Deletion (6)	TGG
	PpKAI2L-B	Insertion (3)	nf
	PpKAI2L-C	Insertion (10) and deletion (15)	nf
I299	*PpKAI2L-A*	Deletion (8)	nf
	PpKAI2L-B	Insertion (7) and deletion (14)	nf
	PpKAI2L-C	Deletion (7)	ACC
I305	*PpKAI2L-A*	Deletion (6)	TGG
	PpKAI2L-B	Deletion (12)	nf
	PpKAI2L-C	Deletion (9)	nf
I410	*PpKAI2L-A*	Deletion (6)	TGG
	PpKAI2L-B	Insertion (1)	nf
	PpKAI2L-C	Deletion (7)	ACC
I415	*PpKAI2L-A*	Deletion (19)	TT
	PpKAI2L-B	Deletion (12)	CTC
	PpKAI2L-C	Deletion (7)	ACC
I417	*PpKAI2L-A*	Deletion (6)	TGG
	PpKAI2L-B	Insertion (10)	nf
	PpKAI2L-C	Deletion (4)	ACC
I421	*PpKAI2L-A*	Deletion (6)	TGG
	PpKAI2L-B	Deletion (12)	CTC
	PpKAI2L-C	Deletion (4)	ACC
I423	*PpKAI2L-A*	Deletion (18)	GCC
	PpKAI2L-B	Deletion (12)	CTC
	PpKAI2L-C	Deletion (5)	CCG
I467	*PpKAI2L-A*	Deletion (3)	nf
	PpKAI2L-B	Deletion (12)	CTC
	PpKAI2L-C	Deletion (5)	CCG

[a]: Genes without mutation (WT) are not shown
[b]:Number of base pairs in brackets
[c]: nf: not found

Table S4 Continued

Mutant Name	Target gene[a]	Type of mutation (bp)[b]	Microhomology motif[c]
I468	*PpKAI2L-A*	Deletion (8)	nf
	PpKAI2L-B	Insertion (30) and deletion (4)	nf
	PpKAI2L-C	Deletion (5)	CCG
I482	*PpKAI2L-A*	Deletion (6)	TGG
	PpKAI2L-B	Deletion (12)	CTC
	PpKAI2L-C	Deletion (5)	CCG
I055	*PpKAI2L-B*	Insertion (1)	nf
	PpKAI2L-C	Deletion (4)	ACC
	PpKAI2L-D	Deletion (11)	nf
I448	*PpKAI2L-B*	Deletion (12)	CTC
	PpKAI2L-C	Deletion (4)	ACC
	PpKAI2L-D	Insertion (6) and deletion (10)	nf
I348	*PpKAI2L-A*	Deletion (17)	nf
	PpKAI2L-B	Deletion (12)	CTC
	PpKAI2L-C	Deletion (6)	nf
	PpKAI2L-D	Insertion (28) and deletion (16)	nf
I405	*PpKAI2L-A*	Deletion (6)	TGG
	PpKAI2L-B	Deletion (12)	CTC
	PpKAI2L-C	Deletion (4)	ACC
	PpKAI2L-D	Deletion (20)	nf
I442	*PpKAI2L-A*	Deletion (6)	TGG
	PpKAI2L-B	Deletion (16)	nf
	PpKAI2L-C	Deletion (5)	CCG
	PpKAI2L-D	Deletion (14)	TCC
I453	*PpKAI2L-A*	Deletion (13)	nf
	PpKAI2L-B	Deletion (12)	CTC
	PpKAI2L-C	Deletion (7)	ACC
	PpKAI2L-D	Deletion (7)	nf

[a]: Genes without mutation (WT) are not shown
[b]:Number of base pairs in brackets
[c]: nf: not found
