# Supplementary material for: Simple and Efficient Targeting of Multiple Genes Through CRISPR-Cas9 in Physcomitrella patens
Source: G3 (Bethesda). 2016 Sep 8;6(11):3647–53. doi: 10.1534/g3.116.033266 (PMC5100863; doi:10.1534/g3.116.033266)
Supplement: Supplemental Material [file supp_g3.116.033266_TableS5.pdf]

Table S5. List of mutants obtained using sgRNAs against *PpKAI2L-A, PpKAI2L-B, PpKAI2L-C, PpKAI2L-D* and *PpKAI2L-E* clade i genes without selection.

Mutant Name	Target gene[a]	Type of mutation (bp)[b]	Microhomology motif[c]
II243	*PpKAI2L-B*	Deletion (12)	CTC
II251	*PpKAI2L-B*	Insertion (14)	nf
II353	*PpKAI2L-B*	Insertion (7)	nf
II364	*PpKAI2L-B*	Deletion (12)	CTC
II421	*PpKAI2L-B*	Deletion (16)	TGT
II478	*PpKAI2L-B*	Deletion (12)	CTC
II484	*PpKAI2L-B*	Insertion (4)	nf
II222	*PpKAI2L-E*	Deletion (9)	nf
II278	*PpKAI2L-E*	Deletion (35)	GCCGG
II328	*PpKAI2L-E*	Deletion (7)	CGGT
II230	*PpKAI2L-B*	Deletion (12)	CTC
	PpKAI2L-C	Deletion (4)	ACC
II502	*PpKAI2L-B*	Deletion (12)	CTC
	PpKAI2L-C	Deletion (4)	ACC
II023	*PpKAI2L-B*	Deletion (12)	CTC
	PpKAI2L-E	Insertion (1) and deletion (11)	nf
II182	*PpKAI2L-B*	Deletion (12)	CTC
	PpKAI2L-E	Deletion (7)	CGGT
II208	*PpKAI2L-B*	Deletion (2)	nf
	PpKAI2L-E	Deletion (7)	CGGT
II218	*PpKAI2L-B*	Deletion (6)	nf
	PpKAI2L-E	Deletion (8)	nf
II257	*PpKAI2L-B*	Deletion (12)	CTC
	PpKAI2L-E	Insertion (4) and deletion (2)	nf
II452	*PpKAI2L-B*	Insertion (30) and deletion (14)	nf
	PpKAI2L-E	Deletion (1)	nf
II087	*PpKAI2L-C*	Deletion (21)	nf
	PpKAI2L-E	Deletion (8)	nf
II224	*PpKAI2L-C*	Deletion (5)	nf
	PpKAI2L-E	Deletion (9)	GG
II274	*PpKAI2L-C*	Deletion (5)	CCG
	PpKAI2L-E	Deletion (8)	nf
II333	*PpKAI2L-C*	Insertion (5)	nf
	PpKAI2L-E	Deletion (8)	nf
II209	*PpKAI2L-B*	Insertion (4)	nf
	PpKAI2L-C	Deletion (7)	ACC
	PpKAI2L-E	Deletion (9)	GG
II457	*PpKAI2L-B*	Deletion (12)	CTC
	PpKAI2L-C	Deletion (7)	ACC
	PpKAI2L-E	Insertion (5) and deletion (3)	nf
II507	*PpKAI2L-B*	Deletion (12)	CTC
	PpKAI2L-C	Deletion (5)	CCG
	PpKAI2L-E	Deletion (7)	CGGT
II239	*PpKAI2L-A*	Deletion (6)	TGG
	PpKAI2L-B	Deletion (40)	nf
	PpKAI2L-C	Deletion (5)	CCG
	PpKAI2L-E	Deletion (28)	nf

[a]: Genes without mutation (WT) are not shown
[b]:Number of base pairs in brackets
[c]: nf: not found
