# Supplementary material for: Simple and Efficient Targeting of Multiple Genes Through CRISPR-Cas9 in Physcomitrella patens
Source: G3 (Bethesda). 2016 Sep 8;6(11):3647–53. doi: 10.1534/g3.116.033266 (PMC5100863; doi:10.1534/g3.116.033266)
Supplement: Supplemental Material [file supp_g3.116.033266_TableS6.pdf]

Table S6. Percentage of frameshift mutations per gene in experiments I to IV targeting *PpKA2L* genes of clade i.

	Percentage of Frameshift (%FS)[b]			
**Target gene[a]**	**Exp. I**	**Exp. II**	**Exp. III**	**Exp. IV**
PpKAI2L-A	44,82	0	n/a	20
PpKAI2L-B	37,93	42,10	n/a	34,88
PpKAI2L-C	95,34	90	92,85	91,42
PpKAI2L-D	83,33	nf	76,47	77,77
PpKAI2L-E	n/a	82,35	65,45	75

[a]: sgRNAs used to target *PpKAI2L* genes are shown in Table S1,
[b]: $\%FS = (\sum FS\ mutations\ by\ \ gene\ in\ the\ exp./\sum mutations\ by\ gene\ in\ the\ exp.)\times 100$
n/a: not applicable, nf= no mutant found
